# Severe Prolonged Hypocalcemia After Denosumab Administration in a Patient With Sleeve Gastrectomy: A Case Report

**DOI:** 10.7759/cureus.106247

**Published:** 2026-03-31

**Authors:** Muhammad J Qassim, Adnan A Gassan, Rehab H Hubail, Mujtaba M Malik, Jenan E Obaid

**Affiliations:** 1 Endocrinology, King Hamad University Hospital, Muharraq, BHR

**Keywords:** bariatric surgery, calcitriol, calcium citrate, denosumab, hypocalcemia, secondary hyperparathyroidism, sleeve gastrectomy, vitamin d deficiency

## Abstract

Denosumab is a human monoclonal antibody that inhibits RANKL, thereby suppressing osteoclast-mediated bone resorption and increasing bone mass in patients with osteoporosis. Although generally well tolerated, denosumab is associated with hypocalcemia, particularly in individuals with impaired calcium and vitamin D homeostasis. We report the case of a 40-year-old woman with a history of sleeve gastrectomy who developed severe, symptomatic hypocalcemia following a single dose of denosumab despite receiving vitamin D supplementation. She presented with neuromuscular symptoms, QTc prolongation, profound hypocalcemia, severe vitamin D deficiency, and marked secondary hyperparathyroidism. Her clinical course was notable for delayed onset, resistance to standard therapy, recurrent hypocalcemia after discharge, and the need for prolonged hospitalization with high-dose intravenous calcium, active vitamin D, and aggressive oral supplementation. This case highlights bariatric surgery as a major and underrecognized risk factor for severe and prolonged denosumab-induced hypocalcemia and underscores the importance of correcting nutritional deficiencies and implementing close biochemical monitoring when initiating denosumab in high-risk patients.

## Introduction

Denosumab is a human IgG2 monoclonal antibody that binds to RANKL with high affinity and specificity. This prevents activation of the RANK receptor on osteoclasts, which are cells responsible for bone breakdown, thereby inhibiting their formation, function, and survival, reducing bone resorption and increasing bone mass and strength [1]. In long-term clinical studies, denosumab has been shown to significantly reduce vertebral and nonvertebral fractures, including hip fractures, and improve bone mineral density [1]. However, rare but serious adverse effects have been reported, including osteonecrosis of the jaw, atypical femoral fractures, malignancies, pancreatitis, and hypocalcemia, which is important as calcium plays a critical role in neuromuscular and cardiac function, and severe hypocalcemia may lead to symptoms such as muscle spasms, seizures, and cardiac arrhythmias [1]. Hypocalcemia has been reported in up to 14% of patients treated with denosumab despite calcium and vitamin D supplementation [2], with a significantly increased risk in those with pre-existing vitamin D deficiency [3]. Vitamin D deficiency is one of the most common nutritional deficiencies observed both before and after sleeve gastrectomy, largely due to impaired absorption resulting from altered gastrointestinal (GI) anatomy [4]. Severe hypocalcemia has been reported following denosumab administration in patients with unrecognized vitamin D deficiency [5]. We present the case of a 40-year-old woman with a history of sleeve gastrectomy who developed severe hypocalcemia following denosumab administration.

## Case presentation

A 40-year-old woman with a history of gastric sleeve surgery four years earlier for bariatric purposes and severe asthma presented to the emergency department in July, 2025, after referral from a local health center for a critically low ionized calcium level of 1.01 mmol/L. She had received a single dose of denosumab eight days prior for osteoporosis, along with vitamin D injections. There was no documented history of prior anti-osteoporotic therapy or fragility fractures. The patient admitted to inconsistent adherence to calcium and vitamin D supplementation following her bariatric surgery. She denied smoking and alcohol use. There was no known family history of metabolic bone disease or calcium disorders. She was not taking proton pump inhibitors or other medications known to impair calcium absorption. No additional significant past medical history was identified apart from severe asthma.

**Figure 1:**
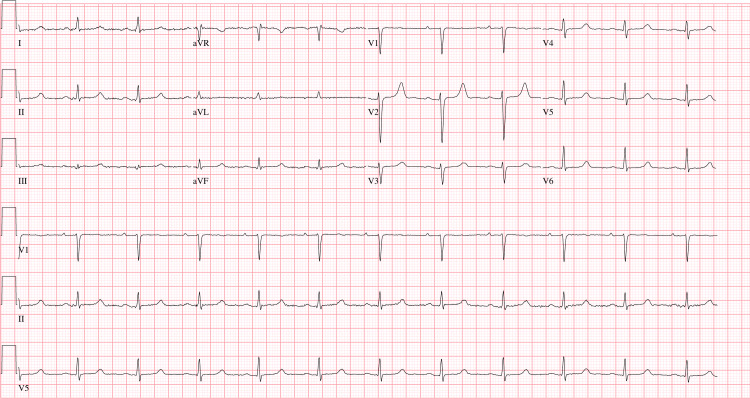
Twelve-lead electrocardiogram demonstrating normal sinus rhythm with prolonged QTc (482 ms) during hypocalcemia

She reported generalized fatigue; bone pain predominantly around the ankles, limiting mobility; perioral numbness; and numbness in her hands and feet, associated with palpitations and shortness of breath. On examination, she appeared clinically stable, with a normal general appearance, and was not in acute distress. She was hemodynamically stable, and cardiovascular, respiratory, and abdominal examinations were unremarkable. However, both Chvostek’s and Trousseau’s signs were positive, with no other focal neurological deficits. An electrocardiogram showed a prolonged QTc interval of 482 ms (Figure 1). Follow-up electrocardiograms after initiation of treatment showed normalization of the QTc interval.

Initial laboratory investigations (Table 1) revealed a measured serum calcium level of 1.04 mmol/L (corrected calcium 1.0 mmol/L), albumin 42 g/L, phosphate 0.7 mmol/L, magnesium 0.69 mmol/L, vitamin D 9.9 ng/mL, and parathyroid hormone (PTH) 969.7 ng/L, consistent with severe secondary hyperparathyroidism due to vitamin D deficiency and hypocalcemia. Serum creatinine and renal function were within normal limits during admission, making chronic kidney disease unlikely. She was also noted to have iron deficiency anemia on admission (hemoglobin 9 g/dL), consistent with the broader nutritional burden in the post-bariatric surgery setting. Liver function tests and blood glucose taken upon admission were within normal limits. She was diagnosed with severe symptomatic hypocalcemia, likely due to denosumab administration in the setting of vitamin D deficiency and malabsorption following gastric sleeve surgery. 

**Figure 2:**
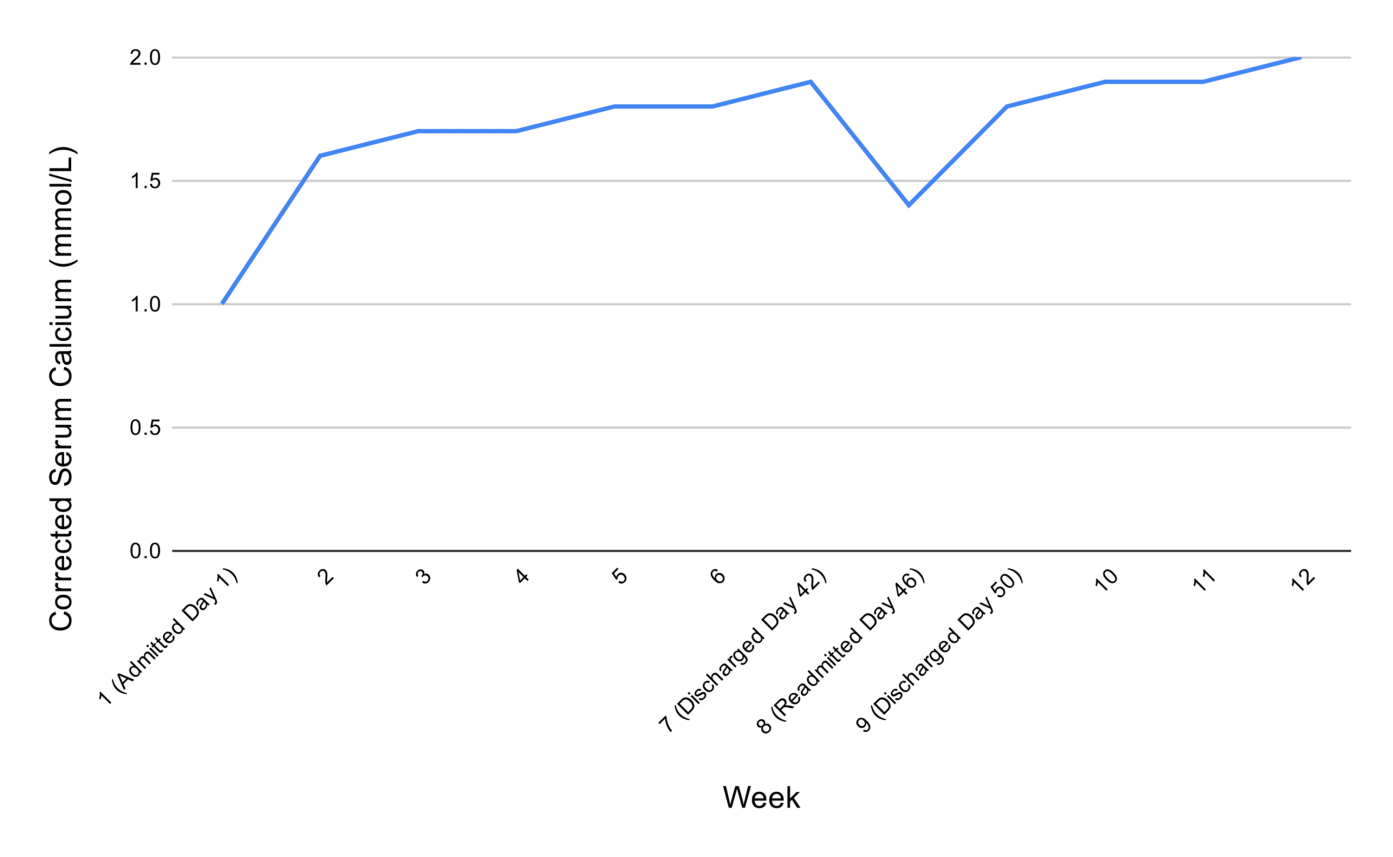
Trend of weekly corrected calcium levels

She was admitted and treated with intravenous calcium gluconate infusions (30-50 mL/hr); a higher rate was not tolerated due to patient discomfort despite weight-based infusion targets for her 60-kg body weight (≈1.5 mg/kg/hour of elemental calcium). High-dose oral calcium carbonate was initially started and later switched to calcium citrate, up to 3 g every four hours. Active vitamin D therapy was initially started with alfacalcidol based on local prescribing practice, with dose escalation from 1 mcg once daily to 3 mcg twice daily, before transitioning to calcitriol up to 3.5 mcg twice daily due to the severity and refractory nature of the hypocalcemia. Cholecalciferol supplementation with 5,000 IU daily was also started and eventually escalated to 50,000 IU twice weekly in response to persistent severe vitamin D deficiency. Thiazide diuretics, up to 25 mg daily, were also added for calcium retention. Mild borderline hypomagnesemia noted on admission was not treated, as it normalized on subsequent testing. Despite aggressive replacement, corrected calcium remained persistently low (Figure 2), necessitating 42 days of inpatient care with frequent adjustments in therapy. Throughout her clinical course, plain radiographs of the chest, sacroiliac joints, and hips were also obtained because of pain, but these were inconclusive and did not reveal a definite acute abnormality.

By Day 42, the patient’s measured serum calcium improved to 2.02 mmol/L (corrected calcium 1.9 mmol/L), PTH decreased to 407.1 ng/L, and vitamin D increased to 53.8 ng/mL (Table 1). She was discharged on the same day with high-dose oral calcium citrate, calcitriol, and vitamin D3 supplementation, but was readmitted four days later with recurrent hypocalcemia (1.46 mmol/L). On further history, she reported running out of calcium citrate and substituting calcium carbonate, after which serum calcium again declined. The recurrence was likely multifactorial, related not only to interruption of calcium citrate supplementation but also to persistent malabsorption and the prolonged pharmacodynamic effects of denosumab. Calcium levels improved only after resumption of calcium citrate and reinitiation of intravenous calcium. She was then discharged on Day 50 on calcium citrate 4 g every four hours, calcitriol 2.5 mcg every eight hours, hydrochlorothiazide 12.5 mg daily, and vitamin D₃ 50,000 IU every other week. She has since maintained stable corrected calcium levels (1.8-2.0 mmol/L) on close outpatient follow-up. 

**Table 1:** Summary of laboratory values

Labs	Values (Day 1)	Values (Day 42)	Reference range
Serum calcium (mmol/L)	1.04	2.02	2.12-2.62
Albumin (g/L)	42	46.5	38-50
Phosphate (mmol/L)	0.7	1	0.8-1.5
Magnesium (mmol/L)	0.69	1	0.7-1.0
Vitamin D (ng/mL)	9.9	53.8	20-80
Parathyroid hormone (ng/L)	969.7	407.1	18.5-88

## Discussion

This report describes the possibility of developing severe hypocalcemia secondary to denosumab administration among bariatric surgery patients, specifically those who underwent gastric sleeve gastrectomy. The effect of such procedures results in impaired calcium solubility and absorption, combined with the pharmacodynamic effects of denosumab, disturbing the homeostasis of calcium. Such an effect could result in a medical complication that could manifest in a complex clinical presentation, leading to a prolonged hospital stay.

In healthy individuals, multiple calcium regulatory systems, such as PTH, the GI tract, the kidneys, and the bones, are present to ensure normocalcemia. However, procedures that manipulate the GI anatomy and physiology, such as bariatric surgery, can dramatically decrease the absorption of calcium [6]. Gastric bypass reduces the stomach size, altering the pH by decreasing gastric acid production, which is essential for calcium solubility and absorption [6,7]. In addition, such procedures reduce the surface area of the duodenum and jejunum, decreasing nutrient absorption significantly [8]. Subsequently, nutritional deficiencies, mainly calcium and fat-soluble vitamins, develop in such patients, predisposing them to severe and symptomatic hypocalcemia and other metabolic diseases [6,9].

Denosumab, a monoclonal antibody against RANKL, inhibits osteoclast-mediated bone resorption and reduces calcium release from bone into circulation [10-13]. Denosumab can be administered subcutaneously, making its absorption independent of GI tract health. This features Denosumab as a superior choice compared to oral medications in post-bariatric surgery patients. However, it is associated with multiple side effects, including hypocalcemia [14]. Multiple studies have reported denosumab-induced hypocalcemia in post-bariatric surgery patients that is associated with poor response to oral calcium and vitamin D supplementation, resulting in a prolonged hospital stay [6,7,10,15,16].

Moreover, it is worth noting that some patients presented with denosumab-induced hypocalcemia several weeks after drug administration. This is due to the pharmacodynamic effect of the drug on osteoclasts, a progressive inhibition of osteoclast activity leading to a prolonged suppression of bone resorption, resulting in a chronic decrease in the release of calcium from the skeletal system to the bloodstream [10,11,17]. Therefore, post-denosumab blood work taken shortly after administration may show normal serum calcium levels. Multiple case studies have reported that denosumab-induced hypocalcemia can take up to four weeks to develop following injection [11,16]. In parallel, PTH levels may rise during this period as a compensatory response to declining serum calcium and vitamin D deficiency. Although PTH would be expected to gradually decrease as calcium and vitamin D levels improve, normalization may be delayed in patients with chronic malabsorption and prolonged calcium depletion, as seen in this case. This emphasises the need for serial calcium monitoring and regular follow-up pre- and post-denosumab administration.

The management of denosumab-induced hypocalcemia in post-bariatric surgery patients is divided into an immediate and long-term phase and requires close monitoring and aggressive supplementation of calcium, vitamin D, phosphorus, and magnesium. In terms of calcium correction, calcium citrate is preferred as it is easily absorbed in low-acidic environments, such as those of post-gastric bypass patients, compared to other forms of calcium. It has been shown that calcium citrate has significantly better absorption, higher bioavailability, and reduced GI side effects in patients with manipulated GI tracts [7].

This case study highlights the effect of denosumab on calcium homeostasis in post-bariatric surgery patients and emphasizes the importance of regular, long-term follow-up with correct supplementation to avoid adverse effects. We recommend that healthcare providers weigh the benefits and side effects of denosumab before prescribing it for patients with altered intestinal absorption. If denosumab is prescribed, it is vital to have serial blood testing and provide calcium supplementation to avoid severe hypocalcemia. Finally, multidisciplinary coordination between primary care providers, endocrinologists, bariatric surgeons, pharmacologists, and social workers is desired to manage and prevent complications that might lead to prolonged hospital stays in such patients. 

## Conclusions

Patients with a history of sleeve gastrectomy are at increased risk of denosumab-induced hypocalcemia due to underlying malabsorption and disruption of calcium-vitamin D homeostasis. This complication may be severe, prolonged, and difficult to manage, particularly in individuals with pre-existing deficiencies. Careful assessment and correction of vitamin D and calcium status prior to initiating denosumab are essential. In post-bariatric surgery patients, ongoing monitoring is warranted, as deficiencies may persist or worsen over time. Given the prolonged pharmacodynamic effects of denosumab, disturbances in calcium balance may also be sustained, necessitating close follow-up. Clinicians should exercise caution when prescribing denosumab in this high-risk population and ensure appropriate preventive and monitoring strategies are in place to minimize complications.
